# Computing the bridge length: the key ingredient in a continuous isometry classification of periodic point sets

**DOI:** 10.1107/S2053273325008253

**Published:** 2025-10-17

**Authors:** Jonathan McManus, Vitaliy Kurlin

**Affiliations:** a Computer Science Department and Materials Innovation Factory, University of Liverpool, Liverpool L69 3BX, UK; Princeton University, USA

**Keywords:** periodic point sets, labelled quotient graphs, isometry invariant

## Abstract

The fundamental model of any periodic crystal is a periodic set of points at all atomic centres. Since crystal structures are determined in a rigid form, their strongest equivalence is rigid motion (composition of translations and rotations) or isometry (also including reflections). The recent classification of periodic point sets under rigid motion used a complete invariant isoset whose size essentially depends on the bridge length, defined as the minimum ‘jump’ that suffices to connect any points in the given set. We propose a practical algorithm to compute the bridge length of any periodic point set given by a motif of points in a periodically translated unit cell. The algorithm has been tested on a large crystal dataset and is required for an efficient continuous classification of all periodic crystals. The exact computation of the bridge length is a key step to realizing the inverse design of materials from new invariant values.

## Abstract 2

We describe an efficient algorithm to compute the bridge length estimating the size of a complete isoset invariant, which classifies all periodic point sets under Euclidean motion.

## 1. Introduction: practical motivations and the problem statement

All solid crystalline materials can be modelled at the atomic level as periodic sets of points (with the chemical attributes if desired) at all atomic centres, defined below.

**Figure 1:**
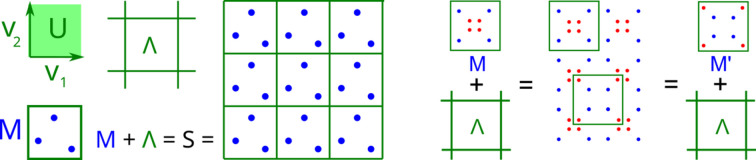
Left: the orthonormal basis 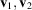 generates the green lattice Λ and the unit cell *U* containing the blue motif *M* of three points. The periodic point set 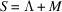 is obtained by periodically repeating *M* along all vectors of Λ. Right: different motifs 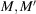 in the same cell generate periodic sets that differ only by translation.

Definition 1 (lattice, unit cell, motif, periodic point set) Any vectors 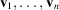 that form a linear basis of 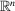 generate the *lattice*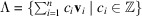 and the *unit cell*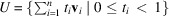. A *motif* is any finite set of points 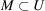, which can represent centres of atoms in a real crystal. The *motif size*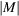 is the number of points in *M*. A *periodic point setS* = 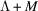 = 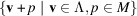 is a union of 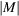 lattices whose origins are shifted to all points *p* of the motif *M* [see Fig. 1 ▸ (left)].

Any unit cell *U* is a parallelepiped on basis vectors 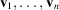. If we translate the unit cell *U* by all vectors 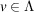, the resulting cells tile 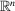 without overlaps. Motif points represent atomic centres in a real crystal. The same lattice can be generated by infinitely many different bases that are all related under multiplication by 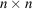 matrices with integer elements and determinant 1. Even if we fix a basis of 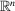 and hence a unit cell *U*, different motifs in *U* can define periodic point sets that differ only by Euclidean *isometry* defined as any distance-preserving transformation of 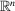.

Since crystal structures are determined in a rigid form, their slightly stronger equivalence is *rigid motion* defined as any orientation-preserving isometry without reflections or as a composition of translations and rotations. After many years of discussing definitions of a ‘crystal’ (Brock, 2021 ▸), a *crystal structure* was recently described in the periodic case as a class of periodic sets under rigid motion (Anosova *et al.*, 2024 ▸).

Any such class consists of all (infinitely many) periodic point sets that are equivalent to each other under some rigid motions. However, almost any perturbation of atoms disturbs some interatomic distances and hence the isometry class with all cell-based descriptors, such as symmetry groups. Even in dimension 1, for any integer 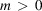 and a small threshold 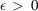, the sequence 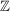 with period 1 is pointwise ε-close to the sequence with the motif 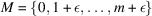 and arbitrarily large period 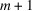.

This inherent discontinuity of all cell-based descriptors was resolved by pointwise distance distributions (PDDs) (Widdowson *et al.*, 2022 ▸; Widdowson & Kurlin, 2021 ▸; Widdowson & Kurlin, 2022 ▸), which defined geographic-style coordinates on the Cambridge Structural Database (CSD) (Widdowson & Kurlin, 2024 ▸). Though PDDs distinguish all periodic crystals in the CSD within minutes on a modest desktop, the only known theoretically complete and continuous invariant that uniquely identifies any periodic point set under isometry in 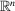 in polynomial time of the motif size (for a fixed dimension) is the *isoset* (Anosova & Kurlin, 2021 ▸; Anosova *et al.*, 2025 ▸).

The invariant isoset requires the bridge length whose definition is recalled below.

Definition 2 [bridge length β(S)] For any finite or periodic set of points 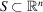, the *bridge length*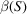 is the minimum distance such that any points 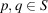 can be connected by a finite sequence of points 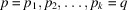 in *S*, such that every Euclidean distance has the upper bound 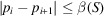 for all 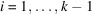.

Equivalently, the *bridge length*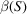 is the minimum double radius such that the union of the closed balls of the radius 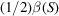 around all points of *S* is connected. The lattice 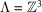 of all points with integer coordinates has 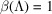. If we add to 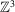 all points whose coordinates are all half-integer, the resulting b.c.c. (body-centred cubic) periodic point set has 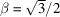 equal to the half-diagonal of the unit cube in 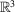.

Expanding Delone’s local theory (Delone *et al.*, 1976 ▸; Dolbilin, 1976 ▸; Dolbilin *et al.*, 1998 ▸; Dolbilin, 2015 ▸; Dolbilin, 2018 ▸), Dolbilin and Bouniaev studied more general *t*-bonded Delone sets, where *t* is an upper bound of the bridge length 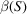 for any periodic point set 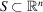 (Bouniaev & Dolbilin, 2017 ▸; Dolbilin & Bouniaev, 2019 ▸). The main problem below is how to create an efficient algorithm to exactly compute 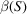.

Problem 3 Design an algorithm to compute the bridge length 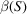 in polynomial time of the motif size for any periodic point set *S* with a fixed unit cell in 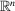.

**Figure 2:**
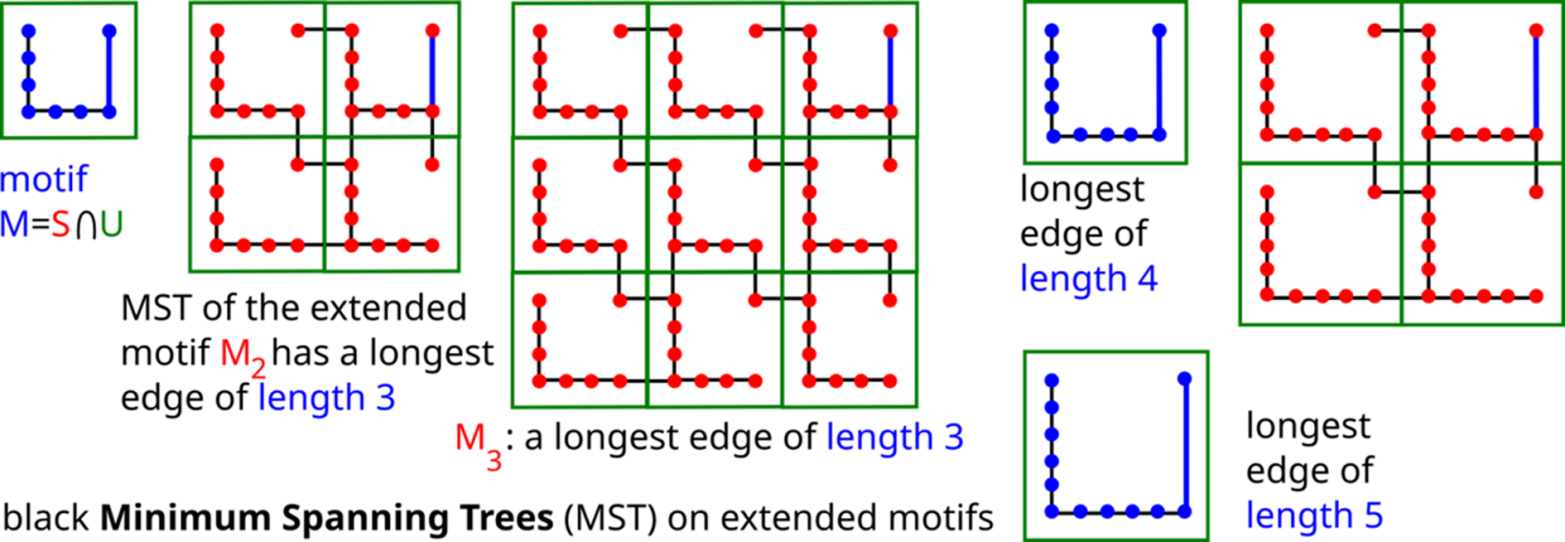
All minimum spanning trees on extended motifs of a periodic point set *S* have the longest edge (in blue) of length 3, which could be made arbitrarily long, relative to a preserved minimum inter-point distance of 1 and bridge length 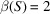 due to shorter edges from the top-right point in every cell across a cell boundary.

The bridge length of a finite set can be computed via a minimum spanning tree but the periodic case does not easily reduce to a finite one, as shown in Fig. 2 ▸.

Definition 4 (minimum spanning tree) For any finite set *M* of points in 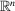, a *Minimum Spanning Tree*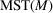 is a tree that has the vertex set *M* and a minimum total length of straight-line edges with lengths measured by Euclidean distance.

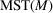
 is uniquely defined if all distances between points of *M* are distinct [see Section 4.3 of Sedgewick & Wayne (2011 ▸)]. By Definition 2[Statement definition2], the bridge length 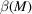 of any finite set 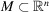 equals the length of the longest edge of 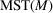.

For any periodic point set *S* with a unit cell *U* on a basis 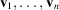 in 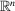, one can consider the extended motifs 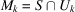, where the extended cell 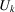 is defined by the basis 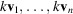 for any integer 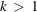. The minimum spanning trees provide the upper bounds 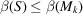 for 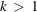, which can be unnecessarily high (see Fig. 2 ▸), so Problem 3[Statement enun3] is much harder for periodic sets than for finite sets of points.

Definition 5 [parameters r(U), R(S), a(U)] Let 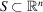 be a periodic point set whose unit cell *U* has a basis 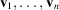. Set 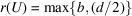, where *b* = 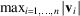 and *d* = 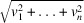. The *covering radius*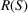 is the smallest radius *R* such that the union of closed balls of radius *R*around all 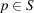 covers 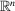. The *height* is 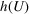 = 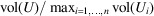, where 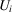 is the subcell of *U* spanned by all basis vectors except 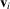. The *aspect ratio* of the cell *U* is defined as 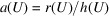.

For any periodic set 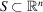, Theorem 2 of Delone *et al.* (1973 ▸) and Lemma 3.6(a) of Anosova *et al.* (2025 ▸) imply the upper bound 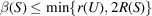, which is too high in practice (see Section 5). Main Theorem 6[Statement theorem6] guarantees an exact computation of 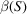 in a time that only quadratically depends on the motif size *m* of *S*.

Theorem 6 For any periodic point set 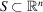 with a motif of *m* points in a unit cell *U*, the bridge length 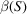 can be computed in time 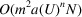, where *N* is the time complexity of the Smith normal form, 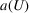 is the aspect ratio from Definition 5[Statement definition5].

As the time complexity is proportional to the aspect ratio 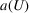 of a cell *U*, an initial reduction of *U* to a smaller cell will speed up the computation of the bridge length by minimizing further cell extensions, namely 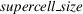 in Algorithm 16[Statement enun16].

Section 2 introduces the key concepts. Section 3 describes the main algorithm for 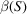. Section 4 proves Theorem 6[Statement theorem6]. Section 5 presents experiments on crystals.

## 2. Auxiliary concepts of graph theory for the bridge length algorithm

This section introduces a few auxiliary concepts to describe the exact algorithm for the bridge length 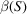 in Section 3 and to prove Theorem 6[Statement theorem6] at the end of Section 4.

**Figure 3:**
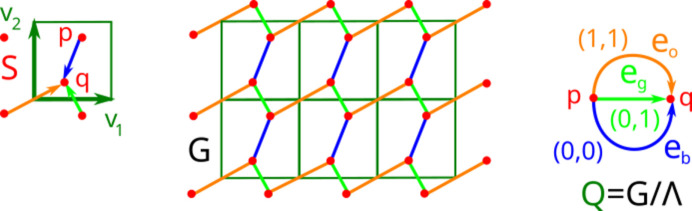
Left: the periodic point set *S* with the basis vectors 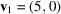, 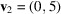 and motif points 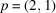, 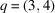. Middle: the periodic Euclidean graph 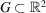 with three types of straight-line edges: green, blue, orange of lengths 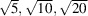, respectively. Right: the labelled quotient graph *Q* has directed edges 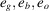 with translational vectors indicating integer shifts of cells (see Definitions 7, 8, 9).

Definition 7 (G\subset{\bb R}^{n}) Let 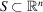 be a periodic point set with a lattice Λ. A *periodic Euclidean graph*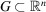 is an infinite graph with the vertex set *S* and straight-line edges such that the translation by any vector 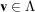 defines an *automorphism* of *G*, which is a bijection 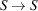 that also induces a bijection on the edges of *G* (see Fig. 3 ▸).

If straight-line edges meet at interior points, they are not considered vertices of *G*.

Fig. 3 ▸ shows a connected periodic graph *G* but *G* can also be disconnected. For example, let *S* be the square lattice 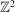, then the graph *G* consisting of all horizontal edges connecting the points 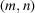 and 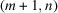 for 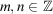 is periodic but not connected. If we add to *G* all vertical edges connecting 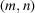 and 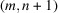 for 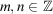, the resulting infinite square grid is a connected periodic graph on 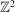.

Definition 8 (quotient graph) Let *G* be a periodic graph on a periodic point set *S* with a lattice Λ in 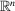. Two points of *S* (also vertices or edges of *G*) are called Λ-equivalent if they are related by a translation along a vector 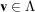. The *quotient graph*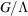 is an abstract undirected graph obtained as the quotient of *G* under the Λ-equivalence. Then *G* is called a *lifted graph* of 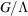. Any vertex of 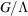 is a Λ-equivalence class 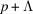 represented by a point 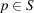. Any edge *e* of the quotient graph 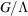 is a Λ-equivalence class 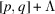 represented by a straight-line edge 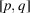 of *G*.

The quotient graph 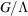 can have multiple edges between the same pair of vertices, as shown in Fig. 3 ▸, which can all be distinguished by the labels defined below.

Definition 9 (labelled quotient graph) Let 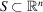 be a periodic point set with a lattice Λ defined by a basis 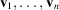. Let *G* be a periodic graph on *S*. For an edge *e* of the quotient graph 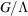, choose any of two directions and a representative edge 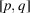 in the lifted graph *G*. Let 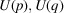 be the unit cells containing 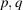, respectively. There is a unique vector 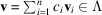 such that 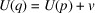 and 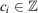, and we define the length of edge *e* in 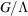 as the Euclidean distance 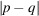. This ‘length’ of an edge *e* is considered an attribute to ease later calculations, and does not change the abstract nature of the quotient graph 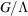.

A *labelled quotient graph (LQG)* is 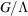 whose every edge *e* has a direction (say, from the Λ-equivalence class of *p* to the Λ-equivalence class of *q*) and the *translational vector*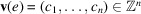 (see Fig. 3 ▸). Changing the direction of *e* multiplies each coordinate of 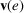 by 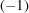. An equivalence of LQGs is a composition of a graph isomorphism and changes in edge directions that match all translational vectors.

Translational vectors 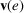 are also called *voltages* if 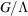 is considered a *voltage graph* or a *gain graph* in topological graph theory. In crystallography, LQGs have been studied by many authors. Chung *et al.* (1984 ▸, Section 6) generated 3-periodic nets by considering LQGs whose translational vectors have entries from 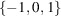. Cohen & Megiddo (1990 ▸, Section 2) described an algorithm to find connected components of a fixed periodic graph in terms of its LQG. Eon (2011 ▸, Proposition 5.1) showed how to reconstruct a periodic graph up to translations from a LQG and a lattice basis, which we also prove in Lemma 10[Statement lemma10] in our notations for completeness. Eon (2016*a* ▸, Section 3) described surgeries on building units of LQGs. Eon (2016*b* ▸, Theorem 6.1) characterized 3-connected minimal periodic graphs (with a slightly different definition of ‘minimal’). McColm (2024 ▸) initiated a search for systematic periodic graphs realizable by real crystal nets (see also Edelsbrunner & Heiss, 2024 ▸).

The LQG 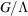 in Fig. 3 ▸ has two vertices 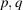. If we orient the three edges of 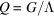 from *p* to *q*, the translational vector 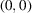 of the blue edge 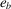 in 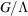 means that the corresponding straight-line blue edge in the lifted graph 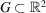 connects points of *S* within the same unit cell *U* with the basis 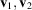. The orange edge with the translational vector 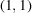 means that each of its infinitely many liftings in 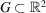 joins a point in a cell *U* to another point in the cell 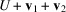.

Lemma 10 (lifting) Let *G* be a periodic Euclidean graph on a periodic point set *S* with a motif *M* in a unit cell *U* defined by a basis 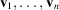 in 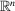. Let *Q* be a LQG of *G*. Then 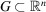 can be reconstructed from *Q*, the basis 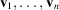, and a bijection between all vertices of *Q* and all points of the motif 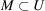.

Proof The basis 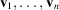 is needed to define a unit cell *U* with the given points of *M*, which are in 1–1 correspondence with all vertices of *Q*. The periodic point set *S*, which is the vertex set of the periodic graph *G*, is obtained from *M* by translations along the vectors 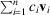 for all 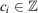. By Definitions 8[Statement definition8] and 9[Statement definition9], every edge *e* of the LQG *Q* has a translational vector 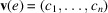 and is a Λ-equivalence class 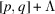 for some 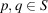 whose unit cells 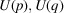 are related by the translation along 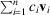. Then we can lift the edge *e* to the periodically translated straight-line edges 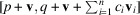 in the periodic graph *G* for all 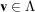.

□

Definition 11 (path/cycle sum) For a path (sequence of consecutive edges) in a LQG *Q*, we make all directions of edges consistent in the sequence and define the *path sum* in 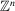 as the sum of the resulting translational vectors along the path. If the path is a closed cycle, the path sum is called the *cycle sum*.

In the language of *voltage graphs*, a path sum may equivalently be referred to as the *net voltage* over the path. In the LQG in Fig. 3 ▸, the upper cycle consisting of the directed orange edge (from *p* to *q*) and the inverted green edge (from *q* to *p*) has the cycle sum 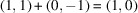. This cycle sum means that a lifting of the cycle to the periodic graph *G* in 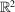 produces a polygonal path connecting a point to its translate by the vector 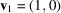 in the next cell to the right.

Definition 12 [minimal tree MST(S/Λ)] For a periodic point set 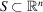 with a lattice Λ, a *minimal tree* is a minimum spanning tree 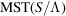 (Definition 4[Statement definition4]) on the set 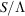 of Λ-equivalence classes of points, where the distance between any classes in 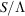 is the minimum Euclidean distance between their representatives in the set *S*.

In Fig. 3 ▸, a minimal tree 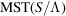 consists of one shortest green edge in 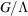.

## 3. Algorithm for the bridge length of a periodic point set

**Figure 4:**
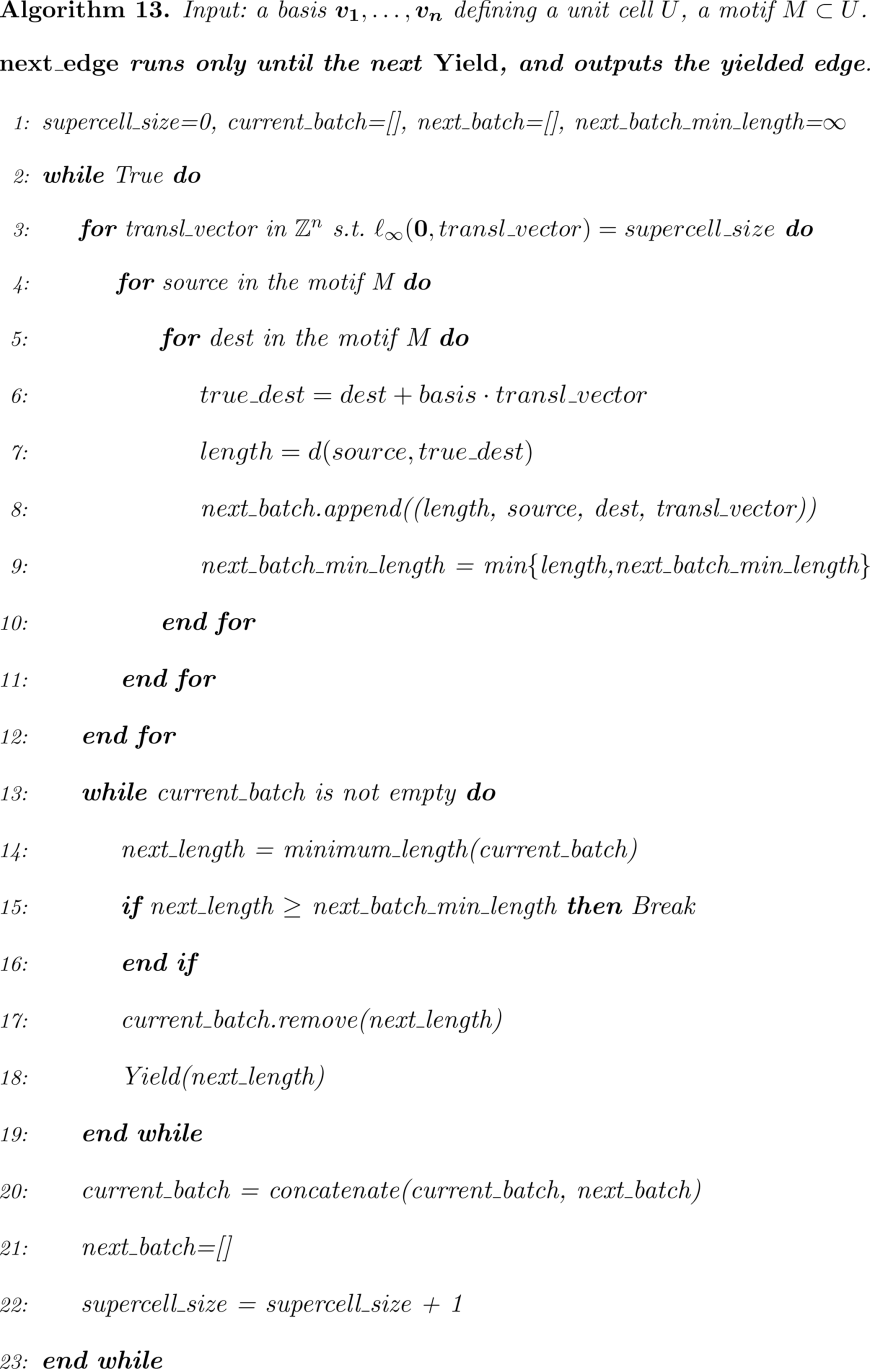
Algorithm 13.

This section will describe the main Algorithm 16[Statement enun16] for solving Problem 3[Statement enun3], which will call auxiliary Algorithm 13 (Fig. 4 ▸) several times. Algorithm 13 starts from a conventional representation of a periodic set 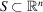 with a motif *M* of points given by coordinates in a basis 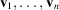 of a lattice Λ, as in a crystallographic information file (CIF).

At every call, Algorithm 13 returns the next shortest edge *e* between points of *S* in increasing order of length. Although *S* is a set of points rather than a graph, we will use the term ‘edge’, because *e* can be considered an edge from a complete graph with the vertex set *S* and with the ‘next shortest edge’ being up to Λ-equivalence.

Any edge *e* between points of *S* will be represented by an ordered pair of points 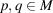 and a translational vector 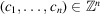 so that the actual straight-line edge in the lifted periodic graph 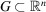 is from *p* to the point 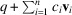. For convenience, we record the Euclidean distance 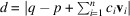 between these endpoints. Then Algorithm 13 outputs any edge *e* as a tuple 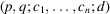.

Algorithm 13 maintains the list of already found edges in increasing order of length. If the next required edge *e* is already in the list, Algorithm 13 simply returns *e*. This shortcut is implemented in Python with the keyword ‘Yield’ – see the documentation at https://docs.python.org/3/glossary.html#term-generator-iterator. Rather than starting from line 1, every time when Algorithm 13 is called, each call ‘Yield *e*’ returns an edge *e*, then temporarily suspends processing, remembering the location execution state including all local variables. When ‘Yield *e*’ is called again, Algorithm 13 picks up where it left off, in contrast to functions that start fresh on every invocation.

If the next edge *e* is not yet found, Algorithm 13 adds more points from a shell of unit cells surrounding the previously considered cells. This *shell* contains the extended motif 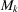 without the smaller motif 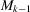 for 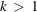 (see Fig. 2 ▸). For any new point *p*, it suffices to consider only edges to points 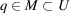 because any edge *e* can be periodically translated by 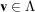 so that one of the endpoints of *e* belongs to *U*. In Algorithm 13, the *Chebyshev distance*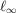 in line 3 is the maximum absolute difference of corresponding coordinates, while *d* in line 7 is the usual *Euclidean distance*.

There is a faster way of checking a condition equivalent to 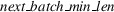 by using the cell geometry. Then, in the vast majority of cases, the algorithm can stop at a supercell one size smaller, which dramatically speeds up the calculation. This calculation is described in Remark 14[Statement enun14]. However, due to the possibility of that not being the case (upon which the algorithm would just default to the same supercell size), we will keep this simpler idea and use it for the time complexity calculations.

Remark 14 (a faster way to compute next\_batch\_min\_len in Algorithm 13) For a unit cell with a basis 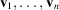, let 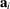 and 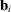 be the shortest vectors parallel and antiparallel to 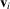 from any point of a motif 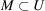 to the opposite boundary faces of the unit cell *U*. Then the faster alternative for 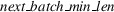 is 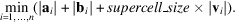 As all the vector lengths 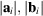, 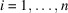 can be pre-computed, we get a massive improvement over the calculation of 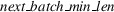 in Algorithm 13.

Algorithm 16[Statement enun16] will be building a LQG *Q* by adding (or ignoring) edges found by Algorithm 13 and monitoring the connectivity of the growing lifted graph *G* whose quotient 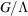 is *Q*. For a basis 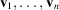 of a unit cell *U* of the lattice Λ of *S*, the edge *e* between points *p* and 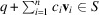 is added to *Q* as the edge between the Λ-equivalence classes of *p* and *q*, with the translational vector 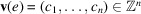. As soon as *G* becomes connected, the length of the last added edge is the bridge length 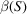, which will be proved in Theorem 26[Statement theorem26] later.

**Figure 5:**
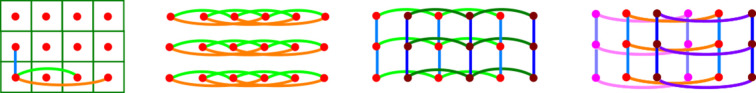
Left: the three vectors 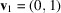, 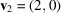, 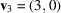 form a basis of 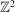. Other images: none of the three pairs 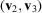, 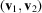, 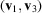 form a basis (insufficient for full connectedness) of 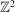. Some straight edges are shown curved for better visibility.

In comparison with a MST of a finite set of points, verifying the connectivity of the lifted periodic graph requires a much more complicated check that translational vectors with integer coordinates form a basis in 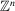 (not 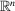), which can include more than *n* vectors. Fig. 5 ▸ shows a basis of 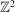 consisting of three vectors, where no vector can be dropped without losing the connectivity of all integer points in 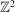.

Algorithm 16[Statement enun16] will use the Smith normal form (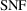) of a matrix of vectors 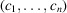 in 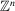 (Newman, 1972 ▸, see p. 26; Cohn, 1985 ▸; Van der Waerden, 2003 ▸, ch. 3.6) for finitely generated modules over a principal ideal domain (PID).

Definition 15 (SNF and invariant factors) For integers 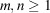, let *A* be a non-zero 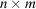 matrix over a PID *P*, for example, 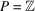. Then there exist invertible 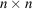 and 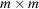 matrices *L*, *R*, respectively, with coefficients in *P*, such that the product *LAR* is an 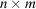 matrix whose only non-zero entries are diagonal elements 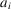 such that 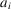 divides 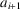 for 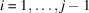, and 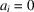 for 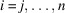 for some 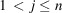. This diagonal matrix *LAR* is the *Smith normal form*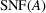. The diagonal elements 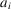 are called the *invariant factors* of *A*.

Let 1 denote the unit element of a PID *P*. If 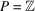, then 1 is the usual integer 1. The simplest SNF has all invariant factors equal to 1, which happens if and only if the last factor 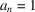 because all previous factors 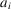 divide 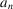.

Algorithm 16 [finding the bridge length β(S) of any periodic point set S\subset{\bb R}^{n}] *Initialization*. A LQG *Q* and a forest 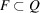 initially consist of *m* isolated vertices, each representing a Λ-equivalence class of a point of the motif of *S*. We will build a *translational matrix A* with columns in 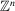, which is initially empty.

*Loop stage*. Consider the next edge 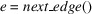 found by Algorithm 13.

*Case 1*. If adding the edge *e* to the current forest *F* would not form a closed cycle (ignoring all edge directions), then add *e* to *F* and *Q* as an edge with an arbitrarily chosen direction and corresponding translational vector 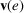 found by Algorithm 13.

*Case 2*. If adding the edge *e* to *F* does form a cycle, find its cycle sum 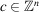 from Definition 11[Statement definition11]. If *c* is not 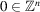 and cannot be expressed as an integer linear combination of the columns from the current translational matrix *A*, then add *e* to *Q* as in Case 1 (but not to the forest *F*) and add the vector *c* as a new column to *A*.

*Termination*. Stop if both conditions below hold, otherwise continue the loop.

(1) The LQG *Q* (hence the forest *F*) becomes connected; and

(2) The translational matrix *A* (whose columns are cycle sums of cycles created by adding edges) has *n* invariant factors equal to 1 (see Definition 15[Statement definition15]).

The necessity of termination condition (1) in Algorithm 16[Statement enun16] means that if the lifted periodic graph *G* is connected, then so is its quotient 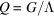. The inverse implication (sufficiency) may not hold. For example, in Fig. 3 ▸, the minimal tree 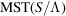 is a single green edge 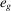, whose preimage under the quotient map 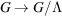 is the disconnected set of all green straight-line edges in the periodic graph 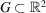.

*Example 17 (running Algorithm 16 on the periodic point set S in Fig. 3)*. The first addition to the quotient graph *Q* and forest *F*, which initially had two isolated vertices 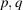, is the shortest green edge 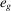 from *p* to *q* (case 1 in the loop stage) with the translational vector 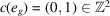. The translational matrix *A* remains empty.

Adding the next (by length) blue edge 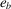 with 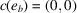 to 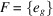 creates a cycle with the cycle sum 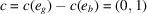. According to case 2 in the loop stage, the quotient graph *Q* becomes the cycle of two edges 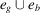 but the forest remains 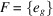. The translational matrix *A* becomes one column 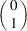
and does not yet have two invariant factors 1. The second termination condition is not yet satisfied, and the current lifted graph consisting of all green and blue segments is still disconnected.

Adding the orange edge 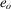 with 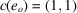 to *F* creates another cycle with the cycle sum 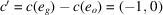. The quotient graph 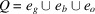 is now full but 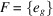 is still one edge. The matrix *A* becomes 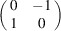
whose SNF is
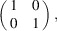
which shows that *A* has two invariant factors equal to 1. Both termination conditions hold and the lifted periodic graph 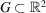 of all green, blue and orange edges is connected. The bridge length 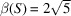 equals the length of the last (orange) edge as expected.

## 4. Correctness and time complexity of the bridge length algorithm

This section proves the correctness of Algorithm 16[Statement enun16] in Theorem 26[Statement theorem26] about the bridge length and main Theorem 6[Statement theorem6] about its time complexity. Lemmas 20[Statement lemma20] and 21[Statement lemma21] will prove the necessity of termination condition (2) in Algorithm 16[Statement enun16]. Both conditions 1 and 2 will guarantee the connectedness of the lifted periodic graph *G* due to Lemma 23[Statement lemma23].

Lemma 18[Statement lemma18] is a partial case of the splitting lemma on p. 147 of Hatcher (2002 ▸).

Lemma 18 (splitting) A short sequence of linear maps 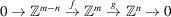 is called *exact* if the image of each map coincides with the *kernel* (subspace mapping to 0) of the next map, *i.e.*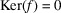, 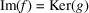, 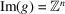. If there is a map 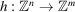, such that 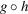 is the identity on 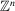, then 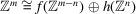, where 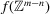 and 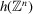 are linearly independent subspaces of 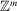 for 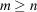.

Note, that, if *f* is a linear map and 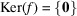, then *f* is injective, because 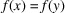 implies that 0 = 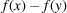 = 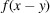, so 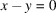 and 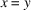.

*Example 19 (finding a SNF)*. In the notations of Lemma 18[Statement lemma18], Fig. 5 ▸ defines 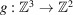 given by the matrix 
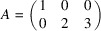
whose three columns generate 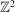. The kernel 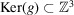 consists of all vectors 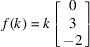
for 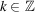, which defines 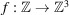 with 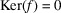 and 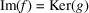 as required in Lemma 18[Statement lemma18]. Since 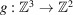 is surjective, we can find a map 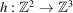 satisfying 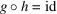, *e.g. h* can be given by 
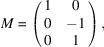
then
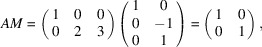
denoted by 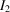. After extending the 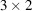 matrix *M* by the extra column with a basis vector of 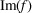, we get the matrix 
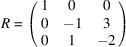
such that 
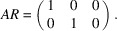


Lemma 18[Statement lemma18] implies that the constituent blocks of *R* are linearly independent to each other; all columns of *R* are linearly independent, and *R* is invertible. Hence, 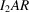 is a SNF of *A* with 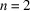 invariant factors equal to 1 by Definition 15[Statement definition15].

Lemma 20 (matrix generating {\bb Z}^{n}\Leftrightarrown invariant factors equal to 1) The columns of any 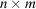 matrix *A* generate 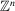 if and only if *A* has *n* invariant factors equal to 1.

Proof Let the *m* columns of *A* generate 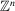. Then *A* defines the surjection 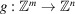 whose kernel 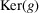 can be obtained as the image of a map 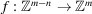, chosen such that 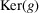 is generated by 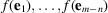, where 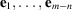 denote the standard orthonormal basis of 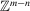. Since 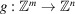 is surjective, orthonormal basis vectors 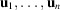 of 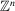 are images 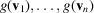, respectively, of some vectors 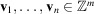. We can define the linear map 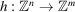, 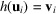 for 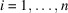, so that 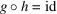 on 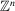. Then *h* has the 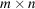 matrix *M* such that 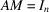, where 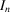 is the 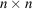 identity matrix. Extending *M* by the 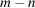 columns 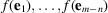 gives the invertible 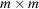 matrix *R* such that *AR* equals the 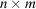 matrix obtained by extending 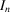 with 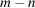 zero columns. Again, *R* is an invertible matrix over 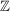, so 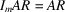 is the SNF of *A* with all invariant factors equal to 1 by Definition 15[Statement definition15].

Conversely, let the Smith normal form 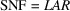 of the matrix *A* in Definition 15[Statement definition15] have all invariant factors equal to 1, so the 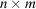 matrix *LAR* has the first *n* columns 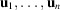, which form a standard basis of 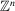, and 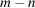 zero columns. Then each 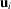 is a linear combination of the *m* columns of *AR* with coefficients from *L*, so these *m* columns generate 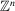. Since *R* is invertible, the *m* columns of *A* also generate 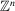.

□

Lemma 21 (connected periodic graph G\subset{\bb R}^{n}\Rightarrown invariant factors equal to 1) In Algorithm 16[Statement enun16], if the lifted periodic graph 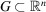 whose vertices form a periodic set *S* becomes connected, then the translational matrix *A* has *n* invariant factors equal to 1.

Proof By Lemma 20[Statement lemma20] it suffices to show that any vector 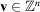 is an integer linear combination of columns of *A*. Let Λ be the lattice of *S* in Algorithm 16[Statement enun16] and 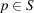 be any point. The points *p* and 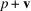 are connected in the lifted graph 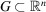 by a polygonal path of straight-line edges. Under 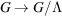, this path projects to a closed cycle *C* at the vertex (Λ-equivalence class) 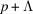 in the quotient graph 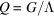.

Let the cycle *C* pass through edges 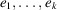 (with integer multiplicities) in the complement 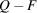 of the forest *F* in the quotient graph *Q*. These edges were added only to *Q* in case 2 of the loop stage. When we tried to add every edge 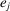 to *F*, the edge 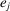 created a cycle 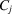 whose cycle sum appeared as a column in the translational matrix *A* (if this cycle sum was not yet an integer combination of the previous columns). Then the vector 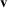 equals the sum of the cycle sums of all the cycles 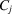 for 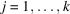, which is an integer combination of the columns of *A* as required.

□

Lemma 22 (connected quotient graph G/\Lambda\Rightarrow\exists a tree of representatives T\subset G) If a LQG 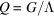 is connected, its lifted graph 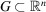 on a periodic point set *S* with a motif of *m* points and a lattice Λ includes a straight-line *tree of representatives*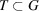 with *m* vertices that are not Λ-equivalent to each other.

Proof Since *Q* is connected, we can choose a spanning tree 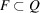 on the *m* vertices of *Q*. A required tree 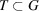 will be a connected union of straight-line edges of *G* that map 1–1 to all edges of *F* under the quotient 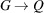. Start from any point 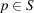 and take any edge *e* at the vertex (Λ-equivalence class) 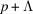 of 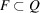. The preimage of *e* under 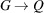 contains a unique straight-line edge 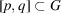, which we add to *T*. After adding to *T* all edges at *p* that project to all edges of *F* at the vertex 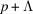, choose another point 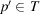 such that the vertex 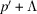 has an edge of *F* not yet covered by *T* under 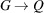. We continue adding edges to *T* by using their projections in 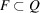 until we get a tree 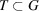 that spans *m* points of *S* that are not Λ-equivalent. The final *T* has no cycle, else this cycle projects under 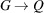 to a cycle in a forest *F*.

□

Lemma 23 (termination conditions in Algorithm 16\Rightarrow connected graph G\subset{\bb R}^{n}) Let *Q* be a LQG with a translational matrix *A* and a lifted graph *G* on a periodic point set 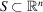 with a lattice Λ. If *Q* is connected and the matrix *A* has *n* invariant factors equal to 1, then the lifted periodic graph 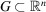 is connected.

Proof For any points 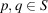, we will find a path of straight-line edges in *G* as follows. By Lemma 22[Statement lemma22] the connectedness of the quotient graph 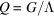 guarantees the existence of a tree 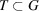 whose vertices represent all Λ-equivalence classes of points of *S*. Let 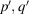 be the vertices of *T* that are Λ-equivalent to 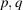, respectively.

Since 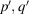 are connected by a path in *T*, it suffices to find a path from *p* to its Λ-translate 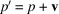 (then similarly from *q* to 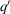) in the graph *G* for any 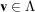. By Lemma 20[Statement lemma20] the columns of *A* form a basis of 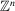, so 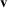 is an integer combination of these columns. It suffices to find a path in *G* by assuming that 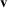 is one column of *A* because a path for any sum 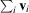 can be obtained by concatenating paths for 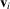. A column 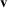 can appear in *A* only in case 2 of the loop stage in Algorithm 16[Statement enun16] as a cycle sum of a cycle 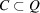 that was created by trying to add an edge *e* from Algorithm 13 to a forest 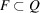. If we order all edges of *C* from the vertex 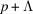 as 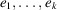, the sum of their translation vectors equals 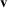. We build a path from *p* to 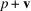 in *G* by finding a unique edge 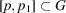 that projects to 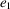, then a unique edge 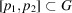 that projects to 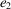 and so on until we cover all 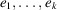 and arrive at 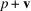.

□

Remark 24 Onus & Robins (2022 ▸) discuss connected components of a periodic graph *K* in terms of homology, namely Theorem 1(1) proves that 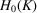 has a basis of 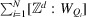 elements, see details in their Section 3.1, but without describing an algorithm for finding such a basis. Our results complement their approach by proving the time complexity for checking the connectivity of a dynamic periodic Euclidean graph in Theorem 6[Statement theorem6] whilst keeping track of its connected components.

Lemma 25 (ignored edges) Let an edge *e* be a Λ-equivalence class of a straight-line edge 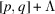 in a lifted periodic graph *G* for some points 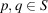. If Algorithm 16[Statement enun16] does not add the edge *e* to a LQG *Q*, then the points 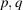 are already connected by a path in the graph 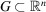 lifted from *Q* by Lemma 10[Statement lemma10].

Proof The loop stage in Algorithm 16[Statement enun16] ignores an edge *e* in the cases below.

Case 1. The edge *e* forms a cycle in *Q* whose cycle sum is the zero vector in 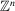.

Case 2. The edge *e* forms a cycle whose cycle sum equals an integer linear combination of pre-existing *cycle sums* from the translational set *B*.

In both cases, we have either one cycle (in case 1) containing *e*, whose cycle sum is 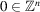, or several cycles (in case 2), one (up to multiplicity) of which contains *e*, whose total sum of translational vectors is 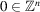. By Definition 9[Statement definition9] each edge of *Q* involved in this zero sum can be lifted to a straight-line edge in the graph 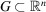.

If we start from the given point 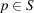, a cycle in *Q* and its sum 0 of translational vectors guarantees that the sequence of the lifted edges in *G* finishes at the same point *p* and hence forms a cycle *C*. This cycle *C* has the edge 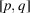 whose exclusion keeps the points 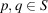 connected by the path in *C* that is complementary to 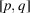.

□

Theorem 26 Algorithm 16[Statement enun16] finds the bridge length 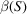 from Definition 2[Statement definition2] for any periodic point set 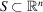 with a motif *M* of points given in a basis 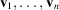.

Proof Within Algorithm 16[Statement enun16], let *d* be the length of the last added edge *e* after which both termination conditions finally hold. By Lemma 25[Statement lemma25] all ignored edges do not create extra connections in the graph *G*. By Lemmas 21[Statement lemma21] and 22[Statement lemma22] the graph *G* obtained before adding the last edge *e* is disconnected. Lemma 23[Statement lemma23] guarantees that, when *e* is added, the graph *G* becomes connected. Because Algorithm 13 yields edges in increasing order, *e* is the shortest edge that could have this property, so the bridge length is 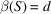.

□

Theorem 6[Statement theorem6] has a rough upper bound assuming that the SNF 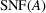 of an integer 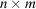 matrix *A* is re-computed for every iteration in time 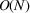. This time was estimated by Giesbrecht (1995 ▸) as 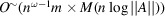, where 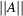 = 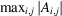 and 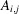 denotes the element of *A* in the row *i* and column *j*. Here 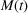 bounds the cost of multiplying two *t*-bit integers, and 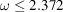 is the exponent for matrix multiplication: two 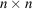 matrices can be multiplied in time 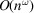 (see Williams *et al.*, 2024 ▸). The ‘soft-Oh’ simplifies the complexity up to logarithmic factors, so 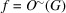 if and only if 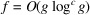 for a constant 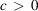.

To speed up Algorithm 16[Statement enun16] in practice, the SNF can be updated at every iteration instead of re-computing from scratch (see details in Appendix *A*[App appa]).

Proof of Theorem 6 Algorithm 16[Statement enun16] solves Problem 3[Statement enun3] by Theorem 26[Statement theorem26]. It remains to show that the time complexity of Algorithm 16[Statement enun16] is 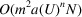. Algorithm 16[Statement enun16] has the initialization of a constant time 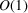 and the loop stage. We will multiply an upper bound for the number of loops by the time complexity of each loop.

One loop in Algorithm 16[Statement enun16] contains at most the following checks:

*(Cycle)* Does adding an edge *e* to a forest *F* create a cycle?

*(Combination)* Is the cycle sum an integer combination of previous cycle sums?

*(Termination)* After appending a cycle sum *c* to the translational matrix *A* and calculating 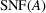, does *A* have *n* invariant factors equal to 1?

The condition *Cycle* is checked by a depth-first search 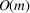 (see Sedgewick & Wayne, 2011 ▸, section 4.1). The condition *Combination* is equivalent to ‘Has 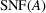 changed?’, and *Termination* is equivalent to ‘Is the product of invariant factors of *A* equal to 1?’. So both conditions can be jointly checked in the time 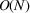 needed to compute 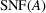.

The time complexity of 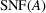 dominates all other steps in Algorithm 16[Statement enun16], so we will use 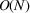 to represent the complexity of a single loop iteration of Algorithm 16[Statement enun16].

Every loop iteration calls Algorithm 13. If we consider all calls to Algorithm 13 as running sequentially, the main loop will run at most 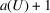 times, where 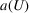 is the aspect ratio from Definition 5[Statement definition5]. By Definition 5[Statement definition5], ‘
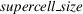
’ must reach at least 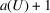 to ensure that we *yield* all potential edges up to and including 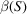, *i.e.*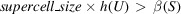. Each loop runs through the unit cells that are ‘
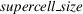
’ away from the central cell 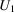. By the end, we will have run through and *yielded* at most 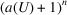 unit cells. For each unit cell 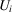, we find all distances between *m* points in 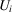 and *m* points in the central cell. The required time is 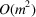 between any two cells and hence 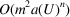 for all cells.

Algorithm 16[Statement enun16] does not run for every edge found by Algorithm 13, but we assume this for simplicity.

The worst-case complexity of this implementation of Algorithm 16[Statement enun16] is 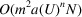.

□

## 5. Experiments on real and simulated crystals, and a discussion

**Figure 6:**
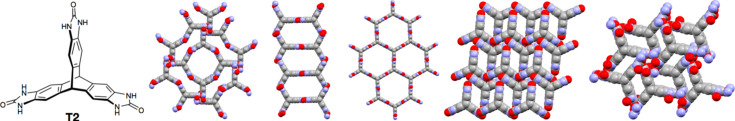
The T2 molecule and five crystals synthesized from T2. The first four T2-α, T2-β, T2-γ, T2-δ were reported by Pulido *et al.* (2017 ▸), the last T2-ε by Zhu *et al.* (2022 ▸).

This section discusses experiments computing the exact bridge length 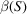 for 5679 simulated and five real nanoporous crystals in Fig. 6 ▸ reported by Pulido *et al.* (2017 ▸).

Table 1 ▸ contains the bridge lengths computed by Algorithm 16[Statement enun16] on the crystals from Fig. 6 ▸ given by their codes in the CSD. The names of the T2 polymorphs refer to the crystalline forms α, β, γ, δ, ε based on the same molecule T2. The crystal IDs in the first column of Table 1 ▸ refer to the CSD six-letter refcodes (Taylor & Wood, 2019 ▸).

Note that there are four slightly different versions of the polymorph T2-γ in the CSD (DEBXIT01…04) because their crystal structures were determined at different temperatures. The seven versions DEBXIT01…07 with the same six-letter code may look similar, even to experts. The polymorph T2-δ (SEMDIA) was deposited later than others because even the original authors confused this polymorph with earlier crystals. This confusion was detected by density functions from the work of Edelsbrunner *et al.* (2021 ▸), computed by Smith & Kurlin (2022 ▸). The underlying density invariants turned out to be incomplete by Example 11 of Anosova & Kurlin (2022 ▸), but were explicitly described for all periodic sequences of intervals in 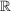 (Anosova & Kurlin, 2023 ▸).

Table 1 ▸ includes the upper bounds 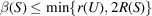 from Lemma 3.6(a) of Anosova *et al.* (2025 ▸) [see 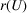 and 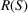 in Definition 5[Statement definition5]]. The run times in Table 1 ▸ were recorded on a laptop with an Intel i5 processor, one 1 GHz core and 8 Gb RAM.

The final row contains the averages for 5679 simulated T2 crystals, which are publicly available in the supplementary materials of Pulido *et al.* (2017 ▸) and were used for predicting the five experimental polymorphs represented by nine entries in the CSD. For all crystals in Table 1 ▸, the translational matrix size never exceeded three columns.

The real T2 crystals in the CSD have smaller motifs consisting of only two or four T2 molecules, while simulated T2 crystals contain up to 32 molecules, which makes the run times slower in comparison with the real ones (see the last column in Table 1 ▸).

More importantly, the exact bridge length 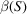 is four times smaller (on average) than its upper bound 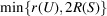. The bridge length 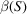 provides the upper bound 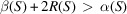 in Lemma 3.6(b) of Anosova *et al.* (2025 ▸) for a stable radius α of atomic clouds that suffices for a complete and continuous isoset invariant of *S*.

This isoset uniquely identifies any periodic crystal *S* under rigid motion and has a continuous distance metric that has detected thousands of near-duplicate crystals. Decreasing the upper bound of 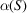 from 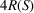 to the smaller value 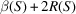 by a factor of about 2 decreases the size *m* of atomic clouds by a factor of 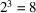 in 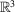. This size reduction speeds up by several orders of magnitude the algorithms for isosets and their distance metric, which have complexity 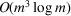 and 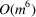 in 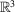, respectively; see the conclusions of Section 5 of Anosova *et al.* (2025 ▸).

The next open problem is an exact computation of the minimal stable radius 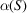. The closely related problem is to compute the *regularity radius* ρ that is the minimum radius with the property that any Delone set with mutually equivalent clusters of the radius ρ is *regular* (periodic with a 1-point asymmetric set) (see Baburin *et al.*, 2018 ▸).

In conclusion, this paper contributes an exact algorithm for computing the bridge length, a key ingredient for solving the geo-mapping problem for periodic point sets within the emerging area of geometric data science (Kurlin, 2025 ▸). This problem has been solved for 2D lattices (Kurlin, 2024 ▸; Bright *et al.*, 2023*a* ▸; Bright *et al.*, 2023*b* ▸), while the 3D case is being finalized (Kurlin, 2022 ▸; Bright *et al.*, 2021 ▸).

## Figures and Tables

**Table 1:** The exact bridge length 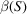 computed by Algorithm 16 and its upper bounds for the nine experimental and 5679 simulated T2 crystals reported by Pulido *et al.* (2017 ▸)

CSD refcodes of experimental and simulated crystals	No. of atoms in a cell	Bridge length 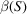 (Å)	Upper bound 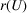 (Å)	Upper bound 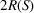 (Å)	Best upper bound over exact 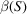	Run time (s)
T2-α NAVXUG	184	2.028	22.325	15.609	7.695	4.337
T2-β DEBXIT05	92	3.163	20.665	12.906	4.080	0.664
T2-β DEBXIT06	92	3.188	20.694	12.884	4.042	0.657
T2-γ DEBXIT01	92	1.879	23.224	23.366	12.358	0.706
T2-γ DEBXIT02	92	1.926	23.226	23.375	12.061	0.636
T2-γ DEBXIT03	92	1.902	23.230	23.373	12.216	0.653
T2-γ DEBXIT04	92	1.970	23.290	23.448	11.824	0.649
T2-δ SEMDIA	92	2.713	14.401	8.350	3.077	0.671
T2-ε DEBXIT07	92	2.062	12.608	5.707	2.768	0.641
						
Average for all 5679 simulated T2 crystals	295.8	2.293	23.306	9.110	4.064	31.653

## Appendix A A faster ‘online’ algorithm for the SNF

A different way of checking the *Termination* condition is to append columns to *A* in an ‘online’ fashion. This avoids the need to calculate the SNF from scratch every time (or often at all), and reduces the complexity to a time close to , where  is the exponent for matrix multiplication (Williams *et al.*, 2024 ▸), and  is the complexity of the extended Euclidean algorithm (Baladi & Vallée, 2005 ▸). As this reduction in complexity is dominated by the price of populating the edges with Algorithm 13, this will be irrelevant for most use cases (and is not used in the experiments shown here). As a use case involves, say, a larger or higher-dimensional pre-populated set of edges, this algorithm becomes more necessary.

Recall from Definition 15[Statement definition15] that the diagonal of the Smith normal form  is made up of the invariant factors of *A*. To progressively calculate , we must only keep track of the right-multiplying unimodular matrix *R*, and the invariant factors themselves, which form a vector . To run the main algorithm here, we do have to begin with a matrix with *n* integer linearly independent rows. ‘Adding’ a vector  to  is where the process changes. We treat *R* and  as mutable, meaning each value is not necessarily fixed to its original assignment. The first step is to define , then we find . If  (*i.e.*), we can continue with , with no need to change *R* as it only keeps track of columns (for context, if we were keeping track of *L* too, we would have to subtract the *i*th row from the last row  times).

If  divides  for all *i*, we would know that including the vector changes nothing; therefore, the relative edge is also irrelevant and can be discarded [this reduces the complexity of most of the *Termination* condition from  to ].

However, if , then  not only becomes , but we also know that  will change and that we must add the edge relative to . We must also alter *R*, accounting for the fact that *F* represents the diagonal of a matrix. We can do this by any process typical of ‘changing the pivot’ in the  algorithm, ensuring that we update *R* in tandem. As accounting for the previous values of *i* is trivial, it is the worst-case equivalent to calculating the  of an  matrix in time , which improves upon the naive calculation of  from scratch upon every alteration of *A*.

Lemma 27 Updating the SNF as above preserves its properties.

Proof As we only alter with elementary row and column operations, this preserves the SNF. By multiplying the to-be-added row  by *R* before concatenating it as a new row to *F*, it is the same as performing those same elementary column operations upon a new matrix:  (*i.e.* concatenated as a row onto *A*). We then continue to perform only elementary row and column operations, and we end up with a matrix that satisfies the conditions of an  noted in Definition 15[Statement definition15]. □

Further discussion of this process is beyond the scope of this paper; however, there are still some small tricks that take advantage of the way the ‘new’ rows for consideration are intrinsically related to , and how  divides .
